# Case Report: A Relatively Mild Phenotype Produced by Novel Mutations in the *SEPSECS* Gene

**DOI:** 10.3389/fped.2021.805575

**Published:** 2022-01-26

**Authors:** Tingyu Rong, Ruen Yao, Yujiao Deng, Qingmin Lin, Guanghai Wang, Jian Wang, Fan Jiang, Yanrui Jiang

**Affiliations:** ^1^Department of Developmental and Behavioral Pediatrics, Shanghai Children's Medical Center, School of Medicine, Shanghai Jiao Tong University, Shanghai, China; ^2^Ministry of Education-Shanghai Key Laboratory of Children's Environmental Health, Xinhua Hospital, School of Medicine, Shanghai Jiao Tong University, Shanghai, China; ^3^Shanghai Center for Brain Science and Brain-Inspired Technology, Shanghai, China; ^4^Department of Medical Genetics and Molecular Diagnostic Laboratory, Shanghai Children's Medical Center, School of Medicine, Shanghai Jiao Tong University, Shanghai, China

**Keywords:** SEPSECS mutation, PCCA, PCH2D, milder phenotype, developmental delay

## Abstract

Mutations in the human *O*-phosphoseryl-tRNA:selenocysteinyl-tRNA synthase gene (*SEPSECS)* are associated with progressive cerebello-cerebral atrophy (PCCA), also known as pontocerebellar hypoplasia type 2D (PCH2D). Early-onset profound developmental delay, progressive microcephaly, and hypotonia that develops toward severe spasticity have been previously reported with *SEPSECS* mutations. Herein we report a case with severe global developmental delay, myogenic changes in the lower limbs, and insomnia, but without progressive microcephaly and brain atrophy during infancy and toddlerhood in a child harboring the *SEPSECS* missense variant c.194A>G (p. Asn65Ser) and a novel splicing mutation c.701+1G>A. With these findings we communicate the first Chinese *SEPSECS* mutant case, and our report indicates that *SEPSECS* mutations can give rise to a milder phenotype.

## Introduction

The *SEPSECS* gene encodes O-phosphoseryl-tRNA:selenocysteinyl-tRNA synthase (SepSecS), the final enzyme that catalyzes the Sep-tRNA to Sec-tRNA conversion, which is used in the synthesis of selenocysteine (1, 2). SepSecS is essential for the synthesis of selenoproteins, which are expressed unevenly and stably in the brain (3). The biallelic deletion of *Trsp* in mice (which impedes selenoprotein synthesis) resulted in cerebellar hypoplasia, seizure, and developmental delay (4)—phenotypes that are congruent with those described in previous clinical reports of *SEPSECS* mutations (5–11). Several investigators identified mutations in human *SEPSECS* as the cause of severe, early-onset neurological symptoms that were later characterized as causing pontocerebellar hypoplasia type 2D (PCH2D) (5–11)—with or without signs of mitochondrial deficiencies that include elevated blood lactate (7), visual impairment, and myopathy (9). Although harboring different mutant alleles, patients presented remarkably similar phenotypes typified by an autosomal recessive progressive microcephaly with profound developmental delay, progressive brain atrophy, and hypotonia (6, 7, 9). Repeated magnetic resonance imaging (MRI) of affected individuals revealed progressive cerebellar atrophy followed by cerebral atrophy involving both white and gray matter. Brain atrophy is recognized at various developmental stages, but principally within the first 18 months of life (6, 7, 9). However, *SEPSECS* mutations have been identified in three milder late-onset patients, with cerebellar atrophy first recognized by MRI at 9, 16, and 18 years of age (8, 10).

We hereby report on a mild phenotype without progressive microcephaly and brain atrophy up to 3 years of age in a Chinese pediatric patient who harbored biallelic *SEPSECS* mutations.

## Case Presentation

### Clinical Data and Laboratory Examinations

**Figure 1:**
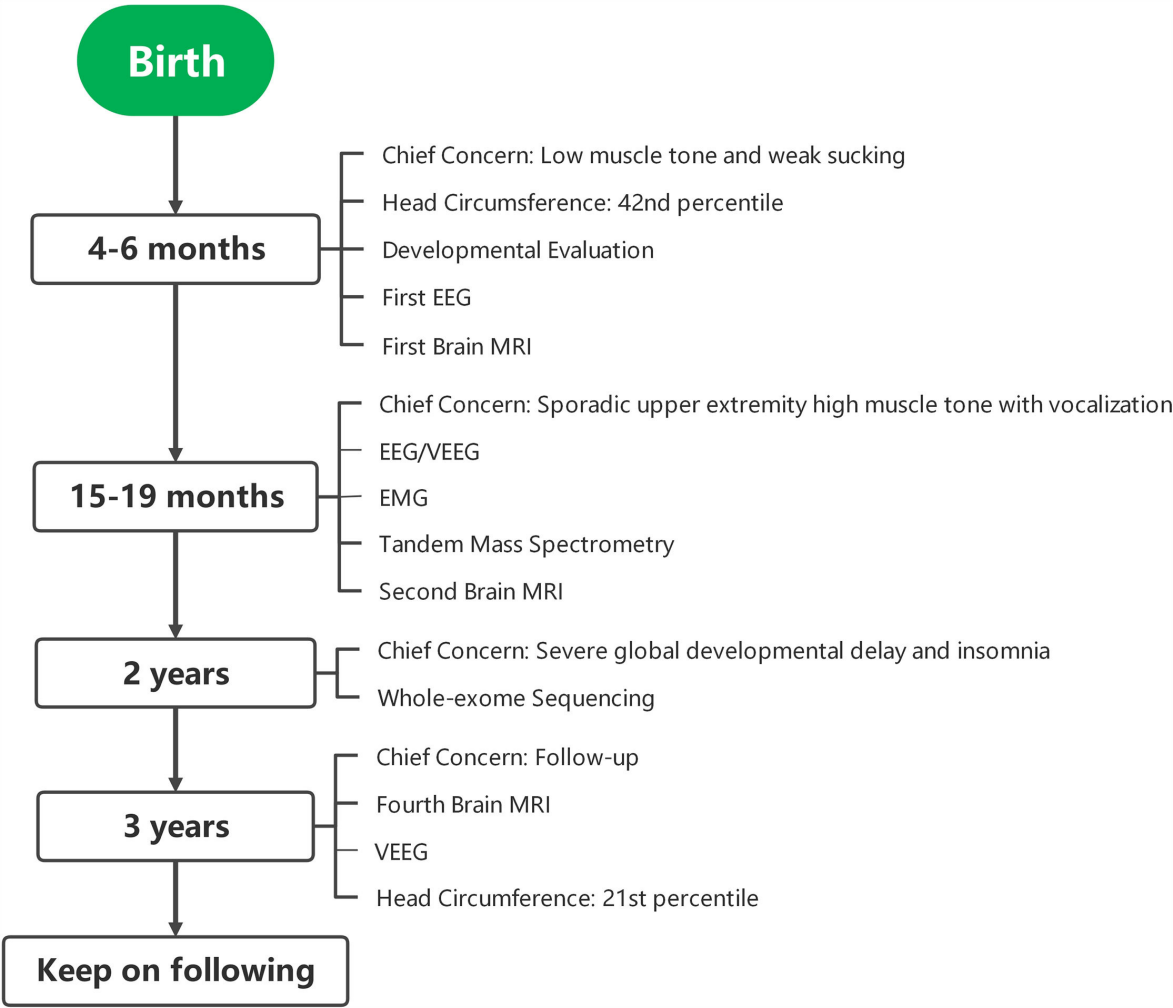
Case timeline. EEG, electroencephalogram; MRI, magnetic resonance imaging; VEEG, video electroencephalogram; EMG, electromyography.

A 2-year-old girl was referred to the Department of Developmental and Behavioral Pediatrics due to severe global development delay and insomnia (case timeline presented in Figure 1). Clinical examination revealed left esotropia, severe muscle hypotonia, and decreased deep tendon reflexes, particularly with respect to both her lower limbs. The patient was born naturally at full-term by healthy non-consanguineous parents. Her birth weight was 3,750 g (86th percentile) and length was 52 cm (94th percentile); head circumference at birth was missing. The patient manifested low muscle tone and weak sucking upon birth, and was still unable to raise her head and turn over her body at 4 months of age. A developmental assessment at 4 months showed that the development quotients of gross motor control, fine motor control, language, reaction to objects, and reaction to people were 55, 66, 38, 28, and 28, respectively. No discomfort or malformation from heart, thorax, kidneys, genitourinary or extremities were mentioned or found. A brain MRI was performed at 5 months, which showed high signal intensities in the bilateral pallidum upon T2W1; her electroencephalography was normal. The patient was subsequently referred to the rehabilitation department for rehabilitation training.

**Figure 2:**
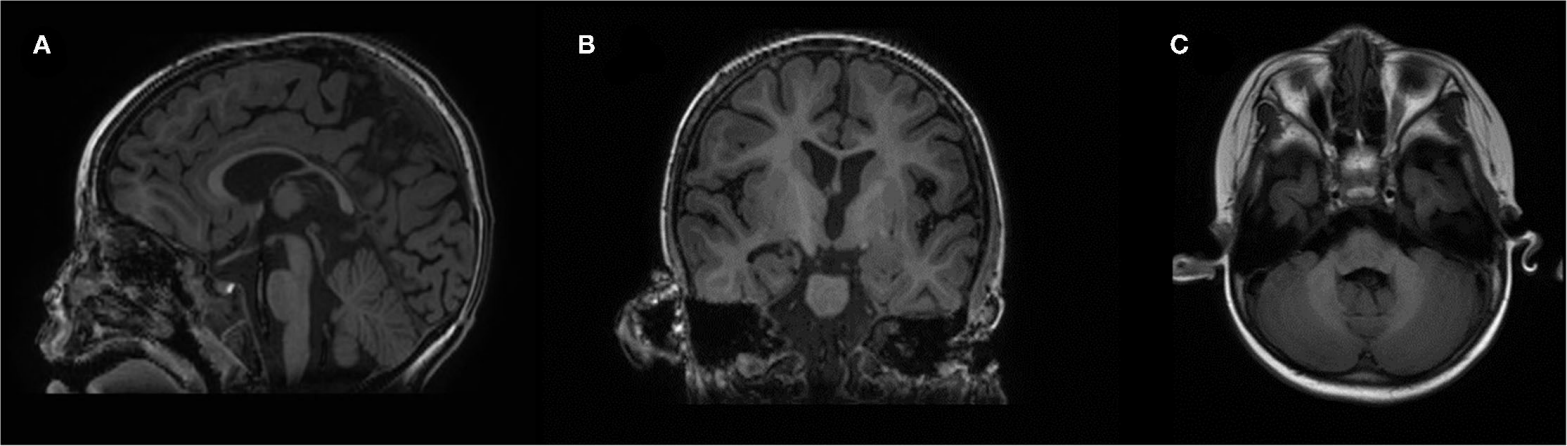
Brain magnetic resonance imaging (MRI): **(A)** sagittal, **(B)** coronal, and **(C)** OAx planes.

At 15 months, the patient was brought to the outpatient unit for sporadic upper extremity high muscle tone with vocalization. Repeated EEG/video electroencephalogram (VEEG) at 15, 19, and 40 months exhibited no sign of epileptic seizure. The second brain MRI at 16 months revealed that the corpus callosum was slightly thin, and the lateral ventricle was plump, and we observed high-plaque signal intensity in the left frontal lobe (Figure 2). Electromyography (EMG) at 18 months showed a slight myogenic change in the tibialis anterior muscle and the peroneal muscle. Tandem mass spectrometry of blood and urine were normal. A follow-up brain MRI at 3 years of age revealed a slight enlargement of the ventricles bilaterally and a deepening of the bilateral frontotemporal sulci. Nevertheless, no obvious atrophy was found upon MRI. Electrocardiograph (ECG) result at 3 years was normal. Available head circumference data were within the normal range (42 cm at 6 months (42nd percentile) and 47.3 cm at 3 years (21st percentile)], as well as her weight [6.9 kg at 4 months (70th percentile) and 16 kg at 3 years (73rd percentile)] and lengths [68.3 cm at 4 months (>99th percentile) and 105 cm at 3 years (96^th^ percentile)] throughout the follow-ups.

### Genetic Testing

**Figure 3:**
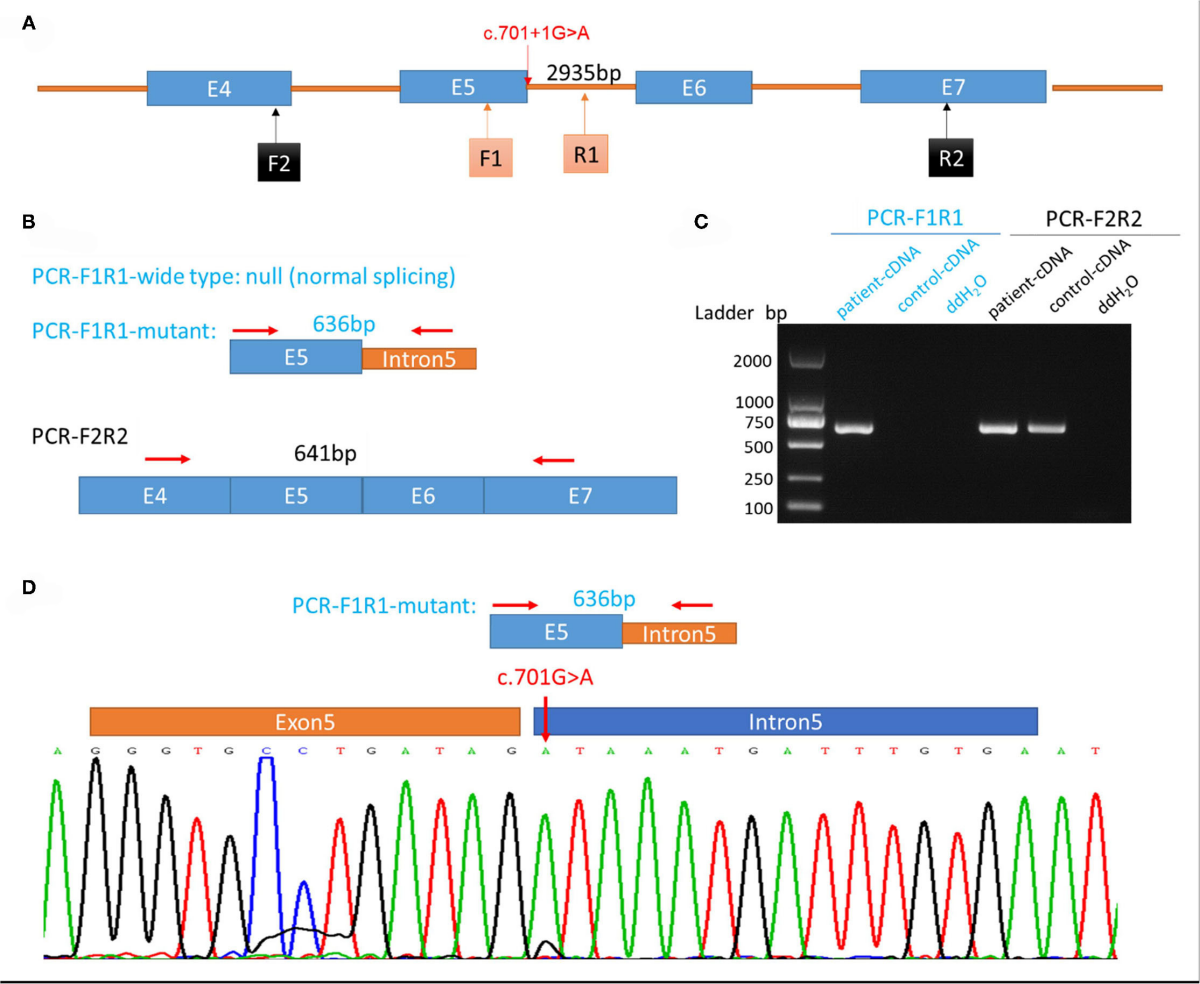
Genetic testing of the patient and analysis of aberrant splicing. **(A)** Schematic diagram of primer design. **(B)** Schematic diagram of expected polymerase chain reaction (PCR) product. **(C)** Electrophoretogram of PCR product from the patient's and control's blood samples. **(D)** Sequence of aberrant splicing product with intron 5 retention (red arrow indicates the mutation site).

Whole-exome sequencing was executed to ascertain a possible molecular cause for the patient's condition. Two variants in *SEPSECS* were subsequently identified: c.194A>G leading to the missense variant p.Asn65Ser, and a novel splice variant c.701+1G>A. Sanger sequencing confirmed these variants and a parental test revealed a compound heterozygous state for the pedigree. To determine the impact of the splice variant, mRNA was extracted from the patient's blood sample for reverse transcription polymerase chain reaction (RT-PCR) assay. We designed the PCR primers as follows: F, 5′-ATCACTGCAGGTTTTGAGCC-3′; R, 5′-ACGCAGACAATGACAACCAC-3′; amplification produced an abnormal cDNA fragment, proving the presence of an alternative splice product with retention of the fifth intron (Figure 3). According to the American College of Medical Genetics and Genomics (ACMG) guidelines for interpreting sequence variants, the c.701+1G>A variant was categorized as pathogenic.

## Discussion

Mutations in *SEPSECS* have now been found in individuals of several races and ethnicities—including Iraqi, Moroccan, Finnish, Arabic, Jordanian, and Japanese (6–9). Herein we reported the first case of pathogenic *SEPSECS* mutations in the Chinese population, which included the pathogenic splice variant c.701+1G>A.

Unlike previously reported cases, the patient in our study did not manifest the typical characteristics of PCH2D, and brain MRI results did not support obvious atrophy. There have been three cases reported of late-onset brain atrophy, either homozygous or heterozygous, and their mutation types are shown in Table 1 (8, 10). The molecular structure of SepSecS includes three domains, two insertions, one pyridoxal phosphate-binding site, and one non-canonical N terminal (1). Van Dijk et al. reported a female patient who exhibited a mild phenotype, with cerebellar atrophy detected at 16 years of age (10). The authors argued that the late-onset phenotype might be the result of a mutation site in the last exon, and this may have allowed partial resumption of enzyme activity (10). Iwama et al. reported two patients with compound heterozygous mutations who shared the same c.356A>G mutation located in the 4th α-helix of SepSecS (8). However, our patient's mutations were located at the 1st insertion and between the 6th β-fold and 5th α-helix. Mutations related to milder phenotypes are located at various sites, and an *in vitro* test of enzymatic activity was not available to us due to technical reasons. Without sufficient evidence to infer prognosis, regular brain imaging and developmental evaluations are still required to determine whether cerebello-cerebral atrophy would occur in the future.

**Table 1:** Summary of *SEPSECS* mutations and related phenotypes.

**Family**	**Ethnicity**	**Sex**	**Gene mutation**	**Protein change**	**Zygosity**	**Brain atrophy**	**Seizure**	**Hypotonia**	**Spasticity**	**Signs of mitochondrial dysfunction**	**References**
1	Chinese	Female	194A>G	Asn65Ser	Compound heterozygous	–	–	+	–	Slight myogenic change in bilateral limbs; muscle biopsy unavailable	Current case
			701+1G>A								
2	Japanese	Female	77delG	Arg26Profs*42	Compound heterozygous	+(9 years)	–	+	+	High blood lactate	(8)
			356A>G	Asn119Ser							
3	Japanese	Female	356A>G	Asn119Ser	Compound heterozygous	+(18 years)	–	+ (3 months)	+ (18 years)	–	
			467G>A	Arg156Gln							
4	Dutch	Female	1321G > A	Gly441Arg	Homozygous	+(16 years)	–	–	–	–	(10)
5,6	Mixed Iraqi-Moroccan	Both sexes	715G>A	Ala239Thr	Compound heterozygous	+(12–28 months)	+ (5 in 7 patients)	–	+ (3–12 months)	–	(5, 6)
			1001A>G	Tyr334Cys							
7	Iraqi	Both sexes	1001A>G	Tyr334Cys	Homozygous						
8,9,10	Finnish	Both sexes	974C>G	Thr325Ser	Compound heterozygous	+(5–6 months)	+ (in 2 of 3 patients at 11–12 months)		+ (within 1 year)	High blood lactate in 2 of 3 patients	(7)
			1287C>A	Tyr429*						
11	Arabian	Male	1001A>G	Tyr334Cys	Homozygous	+(18 months)	–	+ (birth)	+	Borderline abnormal in mitochondrial biopsy; high blood CPK and urinary 3-hydroxyisovaleric acid	(9)
12	Moroccan	Female	114+3A>G		Homozygous	+(4 years)	–	–	+	+	(11)
13	N/A	Female	1A>G	Met1Val	Compound heterozygous	+	+	+	N/A	N/A	(12)
			388+3A>G								
14	Jordan	N/A	1466A>T	Asp489Val	Homozygous	+	N/A	N/A	N/A	N/A	(13)
15	N/A	N/A	1027_1120del	Glu343Leufs*2	Homozygous	N/A	N/A	+	+	N/A	(14)
16	N/A	Male	176C>T	Ala59Val	Homozygous	+	+ (3 weeks)	N/A	N/A	N/A	(15)

*CPK, creatine phosphokinase*.

Epilepsy is a common comorbidity of encephalopathy, and evidence shows that it could exacerbate neurocognitive dysfunction (16). As a result, early diagnosis and treatment of epilepsy are required. Myoclonic or generalized tonic-clonic seizures were previously reported in most *SEPSECS* cases, primarily occurring during the first or second year of life (5, 7, 17). Absence of epilepsy or seizures has also been reported (8–11), especially for milder phenotypes where cerebello-cerebral atrophy was detected in children older than 9 years of age (8, 10). Our patient exhibited seizure-like symptoms at 15 months, as she occasionally manifested upper-extremity high muscle tone together with monotone vocalizations; however, repeated EEG/VEEGs showed no epileptiform discharges at 15, 19, and 40 months. Ruling out epilepsy in our patient was congruent with her milder phenotype. However, with the potential of progressive encephalopathy, additional repeated measurements are essential once symptoms of epilepsy, such as focal or generalized seizures emerge.

Heterogeneous manifestations have been reported in cases with mutations of tRNA synthetase. For example, mutations in *HARS2* (which encodes the histidyl-tRNA synthetase) result in deafness in both genders and infertility in females (18); while mutations in *WARS* (which encodes the tryptophanyl-tRNA synthetase) result in distal hereditary motor neuropathy (19). The heterogeneity in different gene mutations is attributed to the complicated catalytic processes inherent to each tRNA, and the diverse functions of and mechanisms underlying the gene-encoded proteins. *SEPSECS* is essential for selenoprotein synthesis (1); most selenoproteins are oxidoreductases and participate in cellular redox regulation and antioxidant activities (20). Elevated blood lactate levels and visual impairment have been previously reported, suggesting potential mitochondrial impairment due to *SEPSECS* mutations (7–9). Our patient did manifest hypotonia and the EMG showed a slight myogenic change in the lower limbs, although metabolic products in blood and urine remained normal. Muscle biopsy is still an option if further diagnosis is needed.

In conclusion, we have identified a novel pathogenic mutation in the *SEPSECS* gene, which is the first case reported in China, and it is associated with a mild phenotype that encompasses developmental delay, myogenic changes in the lower limbs, and sleep disorder but without progressive microcephaly and brain atrophy. According to other reported cases, disease progression in the future is not precluded, and thus a targeted and detailed follow-up is needed.

## Data Availability Statement

The original contributions presented in the study are included in the article/supplementary files, further inquiries can be directed to the corresponding author/s.

## Ethics Statement

Ethical approval for this study was obtained from the Ethics Committee of Shanghai Children's Medical Center, School of Medicine, Shanghai Jiaotong University (SCMCIRB-W2021030). Written informed consent was obtained from the minor(s)' legal guardian/next of kin for the publication of any potentially identifiable images or data included in this article.

## Author Contributions

TR collected the clinical data and wrote the initial draft of the manuscript. RY determined the mutant gene and participated in writing and reviewing of the manuscript. YD, QL, GW, and JW participated in the diagnosis and critically reviewed the manuscript. FJ and YJ identified and followed up the patient, supervised data collection, and critically reviewed the manuscript. All authors contributed to the article and approved the submitted version.

## Funding

The study was supported by National Natural Science Foundation of China (81773443; 81602868; 82073568; 82071493); Ministry of Science and Technology of China (2016YFC1305203); Science and Technology Commission of Shanghai Municipality (2018SHZDZX05; 18JC1420305; 21Y11907400; 19QA1405800; 19411968800).

## Conflict of Interest

The authors declare that the research was conducted in the absence of any commercial or financial relationships that could be construed as a potential conflict of interest.

## Publisher's Note

All claims expressed in this article are solely those of the authors and do not necessarily represent those of their affiliated organizations, or those of the publisher, the editors and the reviewers. Any product that may be evaluated in this article, or claim that may be made by its manufacturer, is not guaranteed or endorsed by the publisher.
